# Self-Cleaning Biomimetic Surfaces—The Effect of Microstructure and Hydrophobicity on Conidia Repellence

**DOI:** 10.3390/ma15072526

**Published:** 2022-03-30

**Authors:** Haguy Alon, Helena Vitoshkin, Carmit Ziv, Lavanya Gunamalai, Sergey Sinitsa, Maya Kleiman

**Affiliations:** 1 Inter-Faculty Graduate Biotechnology Program, The Hebrew University of Jerusalem, Rehovot 7610001, Israel; haguy838@gmail.com; 2 Institute of Agricultural Engineering, Agricultural Research Organization, Rishon LeZion 7505101, Israel; 3 Institute of Plant Sciences, Agricultural Research Organization, Rishon LeZion 7505101, Israel; 4 Institute of Postharvest and Food Science, Agricultural Research Organization, Rishon LeZion 7505101, Israel; carmit.ziv@volcani.agri.gov.il (C.Z.); biolavanya@gmail.com (L.G.); 5 School of Electrical Engineering, Tel Aviv University, Tel Aviv 6329302, Israel; sinitsa@mail.tau.ac.il; 6 Agro-Nona Technology and Advanced Materials Center, Agricultural Research Organization, Rishon LeZion 7505101, Israel

**Keywords:** bio-inspired, surface microstructure, hydrophobic surfaces, fungus conidia, *Botrytis*, soft lithography

## Abstract

Modification of surface structure for the promotion of food safety and health protection is a technology of interest among many industries. With this study, we aimed specifically to develop a tenable solution for the fabrication of self-cleaning biomimetic surface structures for agricultural applications such as post-harvest packing materials and greenhouse cover screens. Phytopathogenic fungi such as *Botrytis* *cinerea* are a major concern for agricultural systems. These molds are spread by airborne conidia that contaminate surfaces and infect plants and fresh produce, causing significant losses. The research examined the adhesive role of microstructures of natural and synthetic surfaces and assessed the feasibility of structured biomimetic surfaces to easily wash off fungal conidia. Soft lithography was used to create polydimethylsiloxane (PDMS) replications of *Solanum lycopersicum* (tomato) and *Colocasia esculenta* (elephant ear) leaves. Conidia of *B. cinerea* were applied to natural surfaces for a washing procedure and the ratios between applied and remaining conidia were compared using microscopy imaging. The obtained results confirmed the hypothesis that the dust-repellent *C. esculenta* leaves have a higher conidia-repellency compared to tomato leaves which are known for their high sensitivities to phytopathogenic molds. This study found that microstructure replication does not mimic conidia repellency found in nature and that conidia repellency is affected by a mix of parameters, including microstructure and hydrophobicity. To examine the effect of hydrophobicity, the study included measurements and analyses of apparent contact angles of natural and synthetic surfaces including activated (hydrophilic) surfaces. No correlation was found between the surface apparent contact angle and conidia repellency ability, demonstrating variation in washing capability correlated to microstructure and hydrophobicity. It was also found that a microscale sub-surface (tomato trichromes) had a high conidia-repelling capability, demonstrating an important role of non-superhydrophobic microstructures.

## 1. Introduction

Food loss of farm produce during the post-harvest procedures has high economic, environmental, and social costs [1]. Post-harvest food loss of fresh agricultural produce is estimated at more than 30% of total fresh produce [2]. Postharvest food loss may occur for a number of reasons including internal factors, such as enzymatic biodeterioration; or external factors, such as mechanical injuries and pathogenic diseases including yeasts, bacteria, molds, insects, and other organisms [3]. One of the main causes for post-harvest food spoilage are phytopathogenic molds [4]. *Botrytis cinerea* is a necrotrophic fungus that belongs to the *Botrytis* genus [5], among of the most important phytopathogenic fungi [6], and is one of the main causes for postharvest food spoilage [7]. Due to its rapid reproduction and dispersal, resistance to low temperature, and high mutation rate, *B. cinerea* can infect a variety of plant organs and more than 200 plant species, including tomato [8]. Infected plants may show numerous symptoms, including soft rot, collapse and water-soaking of parenchyma tissues, and the appearance of grey masses of conidia, often referred to as the grey mold disease [8]. The ability to thrive in a variety of substrates and environmental conditions—including a wide range of temperatures, water activity levels, and pH values [9]—makes *B. cinerea* a significant pathogen of fresh fruits and fruit juices [10], even in cold storage [11]. The most frequent reproduction mechanism of *B. cinerea* is sporulation [5]. The egg-shaped conidia (spores) are formed on specialized hyphae (termed conidiophores) and are smooth, hyaline or gray, with a mean length of 10 μm and a mean width of 5 μm [12]. Airborne conidia are the most important dispersal propagule of *Botrytis*, which are predominantly wind-dispersed, though water splashes from rain or irrigation can also promote their spreading [13].

Developing new functional surfaces, with fungi repulsion properties, holds great potential for reducing food spoilage. One way of pursuing this is through surface coating. A variety of coatings with chemical additive for food preservative packaging materials, many of which are non-toxic [14,15], or anti-fog coatings [16], as well as self-cleaning coatings [17] for greenhouse covering [18], have been used in recent decades. However, some of the additives can be expensive to produce and integrate, and the regulation process may be very complicated [19]. The main drawback of many coatings is their sensitivity to scratching and degradation [20], especially in non-toxic materials, which are required for food packaging or in greenhouses [21].

Surface structuring is an alternative, chemical-free approach to induce surfaces with different physical properties [22,23,24,25,26]. Many structured surfaces were designed over the years with anti-biofouling properties, reducing the presence of bacteria and biofilms upon the surface [27,28,29]. One example for such a surface showed a significant reduction in Gram-negative bacteria biofouling by applying submicron topographies on biomedical elastomer surfaces. The study compared both single scale nanostructure and multilevel micro and nanostructure to a plain surface, and concluded that in order to achieve the required anti-biofouling response; topographies should not exceed the size of the bacteria [27].

One common inspiration for designing structured surfaces with different properties comes from natural surfaces. This field of natural-based synthetic designs is termed biomimetics [30]. The most known example of such inspirational structure is the hierarchical structure of the lotus (*Nelumbo nucifera*) leaf, which has superhydrophobic and self-cleaning properties [31]. The leaf surface is patterned with micrometric pillars coated with nano-metric wax crystals. Such a structure induces the creation of air cavities between the leaf and a drop of water placed on it, which increases the water/air interface, and minimizes the solid/water interface, and forms a hemispherical drop that rolls off the leaf and carries away dust. Other studies presented superhydrophobic and self-cleaning properties in other natural structures, such as *Colocasia esculenta* leaves structure [32]. Surface engineering may also enhance hydrophobicity through the tailoring of surface roughness [33]. Additionally, other synthetic designs achieved, in some cases, superhydrophobicity and self-cleaning properties based solely on the micrometer scale structure [34,35].

The methods for building such structured surfaces can be classified into two main approaches: building the topography on top of the surface (bottom-up approach) or scraping the surface inwards to create the desired topography (top-down approach) [36]. Some of the techniques that are used in the bottom-up approach include atomic layer deposition [37], sol–gel [38], and vapor phase deposition [39]. There are various methods of lithography that are used for the top-down approach [36,39,40,41]. Although each method has its advantages and disadvantages, soft lithography stands out, especially for replication of biological surfaces due to its cost-efficiency and availability [39,42]. The most widely used polymer in soft lithography is poly(dimethyl siloxane) (PDMS). This material is composed of a moderately stiff elastomer [43], which is non-toxic and biocompatible [44], optically transparent, and well suited for microscopy [45]. Additionally, it is a hydrophobic (water contact angle of ~110°) material that can be modified to hydrophilic (water contact angle can be reduced down to 10°) by exposure to O_2_ plasma treatment [46,47].

Many surfaces, including bioinspired surfaces that are PDMS based, have been studied over the years for anti-biofouling activity. One example is the importance of the surface structure of spinach (*Spinacia oleracea*) leaf in the *E. coli* bacteria attachment, which has been demonstrated by creating a synthetic replication of the leaf surface [48]. In the context of anti-biofouling surfaces, a great example of biomimetic structure is the texture of shark’s skin [24], that was demonstrated in a study that focused on the adhesion of algae zoospores to different textured surfaces. Zoospore settlements were compared between four surfaces, induced with four different microstructures [49]. The study demonstrates a substantial difference between the smooth surface, structured surfaces, and also different geometries of the structures [50]. Considering that most studies were focused on bacteria as the biofouling agent, here we use *B. cinerea* as a representative fungus due to its economic importance, high production rate of average-sized conidia, and its extensive coverage in the literature [51] to design a biomimetic structured surface that repels fungus conidia. We hypothesized that the repelling ability of conidia from the surface can be manipulated through controlling surface microstructure and hydrophobicity. We used two natural leaves as molds for biomimetic structures formed through soft lithography using PDMS. The first is the super hydrophobic leaf of *Colocasia esculenta* (elephant ear) and the second is the leaf of one of *B. cinereas* natural host plants–*Solanum lycopersicum* (tomato). We examined the conidia washing ability of differently structured surfaces with different chemical attributes (hydrophobicity levels) and learned that surface washing ability of conidia is a complex property that does not depend strictly on a single parameter. Additionally, we found that tomato trichomes are microstructural features, which can be replicated to serve as the basis for conidia repelling structured surfaces.

## 2. Materials and Methods

### 2.1. Biological Materials

Leaves of *Colocasia esculenta* (*C. esculenta*) were taken from plants grown for about two years in an outdoor shaded area within a water tank. Leaves of *Solanum lycopersicum* (tomato) from m82 cultivar were taken from plants grown in a growth chamber at 25 °C and under 16:8 long day conditions, fertilized with sapphire nitrate solutions (GAT NPK, Israel). Healthy green leaves, regardless of maturation stage, were chosen for experiments.

*Botrytis cinerea* BO5.10 was grown on potato dextrose agar (Difco, NJ, USA), supplemented with 100 mg/mL chloramphenicol (Fisher BioReagents, Pittsburgh, PA, USA) (PDAC) media, for 10–14 days at 20 °C, until the mature culture produced conidia.

### 2.2. Synthetic Surface Fabrication

The synthetic surfaces (replicas), were fabricated using two-step soft lithography of natural leaves, similar to previously described procedures [48]. Leaves were glued to a petri dish and subjected to dehydration treatment in a desiccator at 80 kPa for one hour. PDMS elastomer and curing agent (SYLGARD 184 kit Dow Chemicals, Midland, MI, USA) were aggressively mixed in a ratio of 10/1 *w/w* respectively, followed by 30 min under vacuum for bubble removal. Polymer was cured in an oven for 20 h, at 45 °C. Leaves were then peeled off carefully using tweezers, and the generated negative molds were submerged in digestion solution (3.5% *w/v* sodium hydroxide and 2.5% *w/v* sodium carbonate, both from Sigma Aldrich, St. Louis, MO, USA, in deionized water) for 10 min, then briefly washed in deionized water and dried at room temperature. Surfaces were then activated using BD-20AC laboratory CORONA treater (Electro-Technic Products, Chicago, IL, USA) for one minute. The negatives were then left in a desiccator at 80 kPa overnight, with 100 µL Trichloro (1H,1H,2H,2H-perfluoro-octyl) silane (Sigma Aldrich, St. Louis, MO, USA), placed on a small plastic plate and left to evaporate to preserve the polymer surfaces at a hydrophilic state. The negative surfaces were used as molds, on which PDMS mixture with similar ratios was poured. Replicas were cured for two days at room temperature until completely firm, then carefully peeled off from the negative molds.

In addition, a flat PDMS surface was prepared using a 10/1 *w/w* ratio of elastomer and curing agent, which were well mixed, followed by a 30 min vacuum for bubble removal. The mixture was then poured into a petri dish until the bottom of the plate was completely covered and cured for two days at room temperature.

### 2.3. Conidia Application and Washing Procedure

Conidia from *B. cinerea* cultures were applied onto natural leaves and synthetic surfaces using dry scattering. Samples were placed on a glass slide, and a petri dish with *B. cinerea* culture was placed upside-down on top of the glass slide and tapped gently several times, to disperse the conidia over the surface of the samples. The samples were then visualized using microscopy (as later described) to generate the image before washing.

For the washing procedure, the tested samples were each placed in a separate Eppendorf tube filled with 1.5 mL DDW and shaken in an MRC Thermo-Shaker DBS-001 (Israel) at 28 °C at 1500 RPM for 5 min. Microscopic images of the surfaces after this step represent the images after washing. For each surface type, 5–11 repeats were performed out of which, at least 3 repeats represented biological repetitions (that is different leaves or replicas of different leaves).

### 2.4. Contact Angles Measurement

Surface contact angle was measured using a KRUSS DSA-100 Drop shape analyzer (Germany). The contact angles of 6 µL water drops were measured with three technical repeats for each surface, at five time points, every 3 s starting at t_0_. The surface contact angle was averaged over all measurements, time points, and repetitions for each surface.

### 2.5. Microscopy and Image Processing

For light microscopy images, Nikon Eclipse Ni, was set to transmitted light, bright-field filter at 10× magnification, with the condenser lens elevated all the way up. Images were taken using a Nikon DS-Ri2 camera and NIS Elements software. All samples were imaged using real-time extended depth focus (EDF) for multi-level focus imaging and auto-exposure setting. Due to space limitation and zooming capabilities, several images were taken per sample, with an overlap of ~20–30% for full coverage. Image stitching algorithm for reconstruction of the whole surface was adopted combining the scale-invariant feature transform (SIFT) [52] algorithm and homography transformation (HT) [53]. For a specific set of images, the SIFT algorithm finds common points in a large number of complex surface textures. Points were used for the calculation of a homography matrix for each pair of images. Once the matrices were calculated, the pairs of images were transformed and combined.

A manual conidia count was performed using manual labeling of conidia on a stitched image by a single person, using LabelImg software v1.8.1. Labels were saved in extensible markup language (XML) format. Labels were counted, and results were summarized in a table and exported as an Excel file. All algorithms for mentioned applications were written in Python with OS, LXML, Pandas [54], CV2 [55], Matplotlib [56], and Numpy [57] modules. The area of each sample was measured from the sample stitched image using ImageJ software 1.53e.

All SEM images were taken using a JEOL-benchtop JCM-6000 (Tokyo, Japan) scanning electron microscope. Before imaging, natural leaves were washed briefly with deionized water to remove dirt particles and then placed in small, sealed bottles, one leaf in each bottle. Leaves were covered with 50% ethanol solution and left for one hour at room temperature. Ethanol solution was replaced every hour with increasing concentrations of ethanol (70%, 90%, 95%, 100%). Leaves were kept for at least two hours and up to three days in 100% ethanol at room temperature before critical point drying (CPD) with K850 Quorum Critical Point Dryer. Synthetic replications and dehydrated natural leaves were coated with a thin layer of gold using a Quorum SC7600 mini sputter coater.

### 2.6. Statistical Analysis

The statistical analysis and graphic visualization were carried out using the Statsmodels [58], Scipy [59], Pandas [54], and Seaborn [60] modules of Python 3.8 software. The data for the analysis includes conidia repellency values of each sample (conidia count before/after wash), contact angle measured on all surfaces, the material of the surface (natural leaf, PDMS, activated PDMS), the structure of the sample (col, tom, flat), the total number of conidia on the sample before wash and the date of experiment. The number of conidia on the trichomes and the number of conidia on the rest of the surface of the sample before and after wash were counted separately for each sample of synthetic tomato leaf replication (PDMS and activated PDMS). Replicated leaf trichomes were counted for each synthetic (PDMS and treated PDMS) sample. Conidia repellency levels of trichomes and of the rest of the surface were calculated separately using the same calculations. Conidia repellency of various surfaces was compared using a Student’s *t*-test for equal variances. The contact angle of different surfaces was compared using pairwise Welch *t*-tests for unequal variances. A pairwise Student’s *t*-test for equal variances was also used to compare the conidia repellency of sub-structures of synthetic replications: trichomes and the rest of the surface. Homogeneity of variances was established by the Levene test using the Python algorithm. Correlations between conidia density before washing to the washing efficiency level of samples were tested for each surface using Pearson’s correlation tests, and a one-way ANOVA test was used to assess the effect of the date of the experiment on the washing efficiency of surfaces. Results with a significance level of *p* < 0.05 were considered significant. For all multiple pairwise *t*-tests, α was adjusted using Bonferroni correction.

## 3. Results

### 3.1. Conidia Repellent Characteristics of Natural and Synthetic Systems

**Figure 1:**
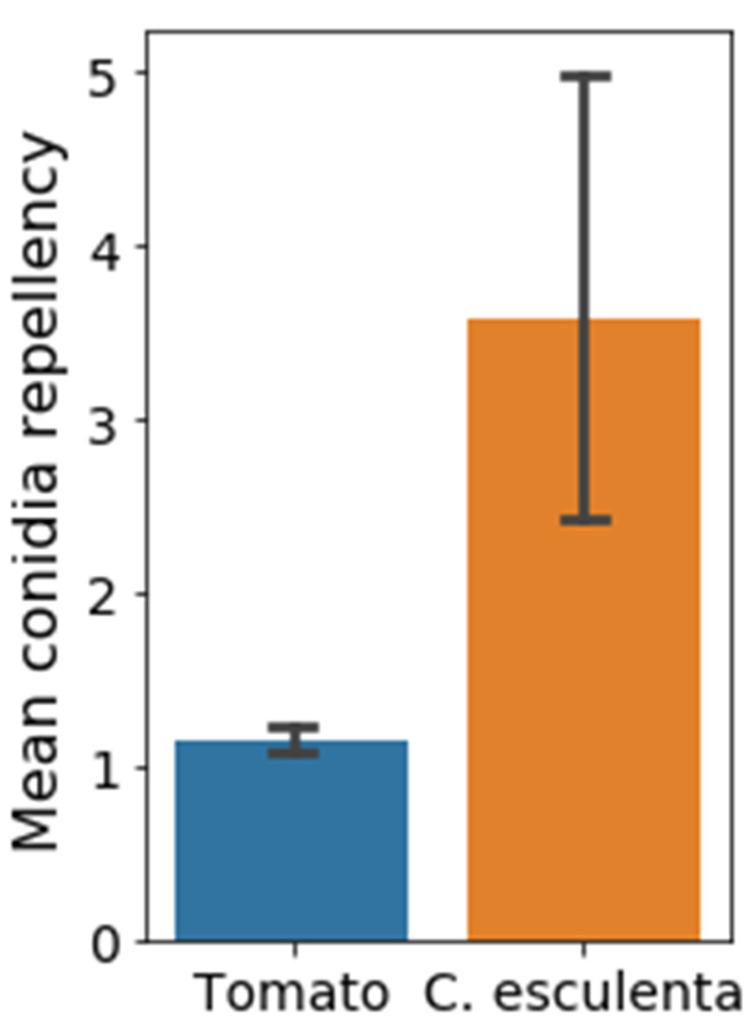
Conidia repellency of two natural surfaces. *C. esculenta* leaves and tomato leaves were tested for their conidia repellency. Conidia repellency represents the number of conidia counted before wash, divided by the number of conidia counted after wash (BW/AW) on the same surface. Pairs comparison was conducted using Student’s *t*-test. Conidia repellency values of *C. esculenta* leaves (3.57 ± 0.69) were significantly higher than those of tomato leaves (1.15 ± 0.04) (*p* < 0.01, Bars represent STD).

To compare the efficiency of conidia wash from different surfaces, a specific washing procedure was calibrated using *B. cinerea* conidia and different surfaces, as described in the Materials and Methods Section). Conidia repellency is a unit less number that was determined as the ratio between the number of conidia counted before wash (BW) to the number of conidia counted after wash (AW), on the same surface (BW/AW). We hypothesized that *C. esculenta* leaves, which are known for their self-cleaning and dust repellency properties, will show high conidia-repellency. Therefore, conidia repellency values of *C. esculenta* leaves were compared to those of leaves from tomato, host plant of *B. cinerea*, and a crop that suffers great economic damages due to phytopathogenic molds. As expected, the mean conidia repellency of *C. esculenta* leaves was higher compared to that of tomato leaves (Figure 1).

**Figure 2:**
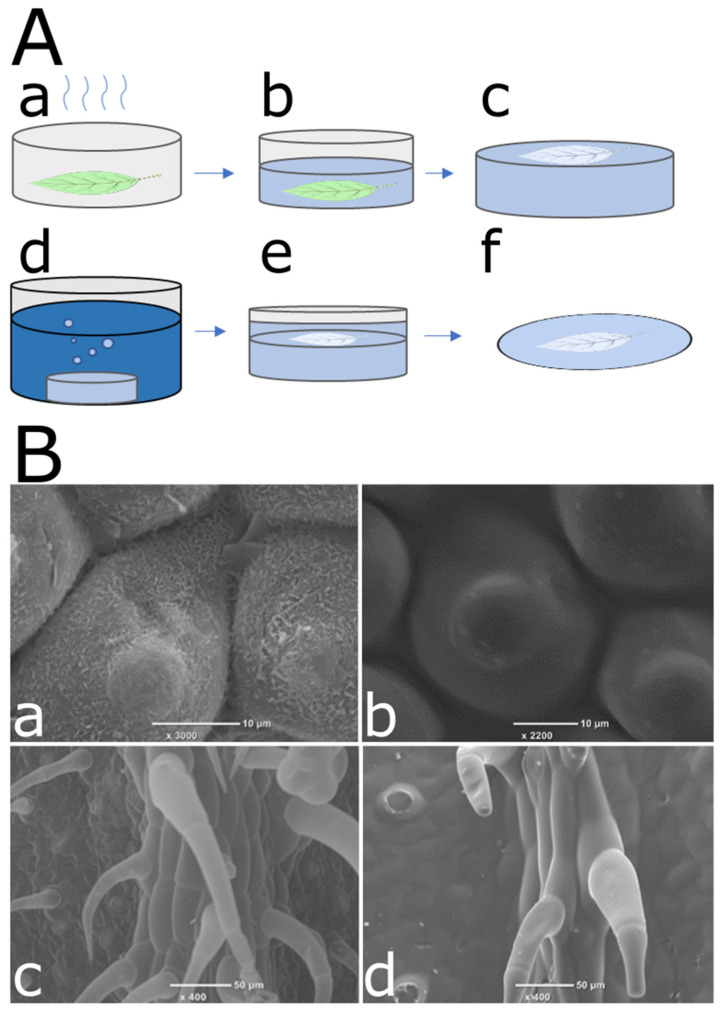
Replication of *C. esculenta* and tomato leaves microstructure. (**A**) Schematics of the replication process. A leaf was glued to a petri dish and dehydrated (**a**). Soft polymer was applied onto the leaf and cured in an oven (**b**). The leaf was carefully peeled off the negative mold (**c**). Negative mold was submerged in a digestion solution, and any leaf traces were removed (**d**). Negative mold was dried and treated with plasma, and then soft polymer was applied to the mold (**e**). Leaf replica was cured at rooms temperature and carefully peeled off the negative mold (**f**). (**B**) SEM micrographs of *C. esculenta* leaf (**a**) and its PDMS replication (**b**), tomato leaf (**c**) and its PDMS replication (**d**). Leaves were fixated and underwent CPD, as described in the methods section. The leaves were then coated with a thin layer of gold and visualized using SEM at a high resolution. Replicas were coated and visualized by SEM. The images show good replication quality of leaf microstructure.

**Figure 3:**
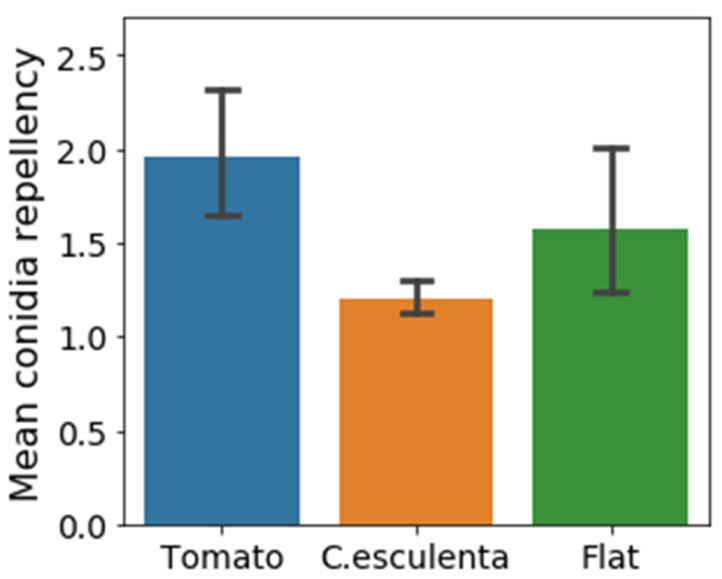
Conidia repellency ability of synthetic surfaces. Conidia repellency values of three PDMS surfaces: *C. esculenta* and tomato leaf replicas (orange and blue respectively) and flat surface (green) as a control for a structure less surface with the same chemical properties. Pairs comparison was conducted using Student’s *t*-test, α was adjusted using Bonferroni correction. The microstructure of tomato leaves seemed to induce significantly higher conidia repellency values (1.9 ± 0.18) than the texture of *C. esculenta* leaves (1.2 ± 0.05) (*p* < 0.01).

In the next step, we examined the correlation between conidia repellency and surface microstructure since surface microstructure is a crucial part of the functionality of many surfaces, specifically in biological systems. *C. esculenta* and tomato leaves vary in many parameters, including chemical composition and microstructure. To assess the structural contribution to the high conidia repellency observed in *C. esculenta* leaves, natural leaf microstructure was replicated in a synthetic system. Replications were fabricated using soft lithography and the commonly used polymer, polydimethylsiloxane (PDMS). The two-step replication process is illustrated in Figure 2A. Figure 2B presents SEM images of *C. esculenta* and tomato leaves as well as their replicated microstructure, showing a reliable replication of micro but not nano structures. The lack of nano-structure replication is due to our choice to use only inexpensive replication methods since we are seeking an affordable solution for surface fabrication. Conidia repellency of *C. esculenta* and tomato leaf replicas, as well as flat PDMS surfaces, were compared to evaluate the effect of microstructural modifications on the surfaces conidia repellency ability (Figure 3). Contrary to our initial hypothesis, the synthetic system did not replicate the trend we observed in the biological system. Conidia repellency of tomato leaf replicas was significantly higher than that of *C. esculenta* replicas, while flat surfaces showed intermediate conidia repellency values that did not differ significantly from *C. esculenta* nor tomato leaf replicas (Figure 3).

### 3.2. Microstructure, Hydrophobicity, and Conidia Repellency

**Figure 4:**
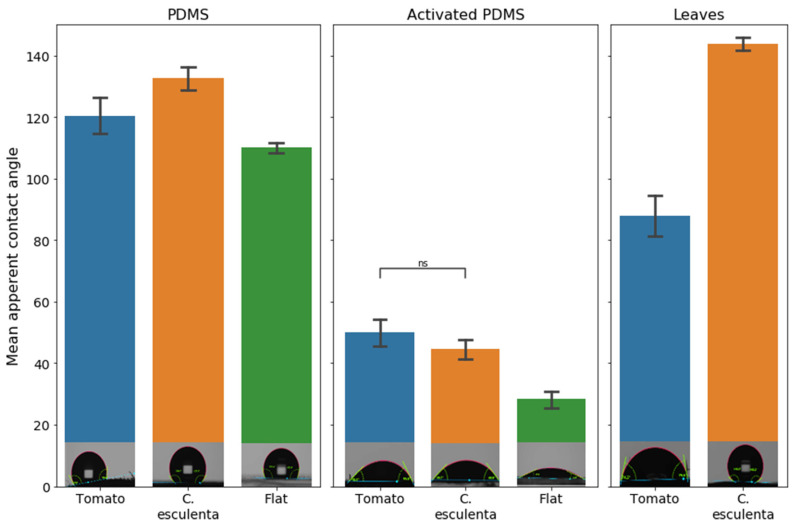
Hydrophobicity measurements of natural and synthetic surfaces. The mean apparent contact angle of synthetic and natural surfaces was measured. The bottom row presents images of the drop in a stable state. The drop spreads on hydrophilic surfaces (for example, surfaces in the middle section) and does not do so on hydrophobic surfaces (for example, surfaces in the left section). Pairs comparison was conducted using Welch’s *t*-test, α was adjusted using Bonferroni correction. The mean contact angle of PDMS replicas is significantly higher than the mean contact angle of activated PDMS surfaces (*p* < 0.001) for all structures under consideration. The surface contact angle of synthetic surfaces (PDMS, activated PDMS) are significantly larger than chemically identical flat surfaces (flat) (*p* ≤ 0.04). Contact angle measured on *C. esculenta* leaf (143.7° ± 4.4°) is significantly higher than the contact angles measured on tomato leaf (87.9° ± 13.3°) with *p* ≤ 0.00001.

The relatively low conidia repellency values demonstrated by the *C. esculenta* leaf replica indicated that properties other than microstructure are involved in the conidia-repellency of *C. esculenta* leaves. As the self-cleaning properties of *C. esculenta* leaves are often correlated to their superhydrophobicity [32], we measured the apparent contact angle of the tested surfaces to inspect their hydrophobicity and a possible correlation between this property and conidia repellency. Figure 4 presents the measured mean apparent contact angle of synthetic microstructured replicas and natural surfaces. As expected, the mean apparent contact angle of the super hydrophobic *C. esculenta* leaf surface is significantly higher than that of the tomato natural leaf surface. However, the apparent contact angle of the PDMS microstructure replica of *C. esculenta* leaf is only slightly higher than the contact angle of the PDMS replica of tomato leaf. This is because the chemical basis of both surfaces in the synthetic system is identical (as opposed to the natural system) and the change in apparent contact angle results only from the change in microstructure. Hence, the *C. esculenta* leaf microstructure shows a more hydrophobic nature than tomato leaf microstructure, as expected, though to a lesser extent than in the natural system.

The apparent contact angle was also measured on PDMS surfaces with similar microstructures, but which were activated to turn the surface chemical properties more hydrophilic, using O_2_ plasma, as described in the methods section. The results show that the mean contact angle of PDMS microstructure replicas is significantly higher than those of activated PDMS surfaces for all structures under consideration, as expected. Additionally, structured surfaces demonstrated a larger apparent contact angle than chemically identical flat surfaces in both hydrophilic and hydrophobic PDMS surfaces. Moreover, in hydrophobic PDMS surfaces, the apparent contact angle measured on *C. esculenta* leaf replica was larger than on tomato leaf replica, while in hydrophilic PDMS surfaces, it was slightly lower. This shows that both chosen microstructures cause an increase in surface hydrophobicity, the extent of which depends on the surface chemical properties.

**Figure 5:**
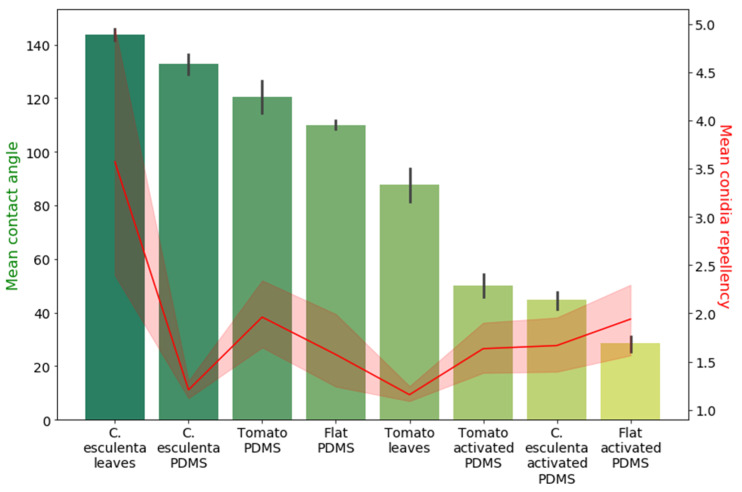
Surface hydrophobicity and conidia repellency. A comparison between the mean contact angle (green columns) and the mean conidia repellency (red line) of natural and synthetic surfaces. No correlation was found between the two properties.

At this point, we wanted to correlate surface hydrophobicity and conidia repelling values. To do so, we tested all surfaces, including the activated surfaces, for their conidia repellency. The correlation between the mean apparent contact angle of all surfaces with their spore repellency is demonstrated in Figure 5. No correlation was found between the surface apparent contact angle and conidia repellency ability, suggesting that hydrophobicity cannot act as the main mechanism explaining the differences in conidia repellency between the surfaces.

### 3.3. Sub-Surface Microstructure Conidia Repellency Abilities

**Figure 6:**
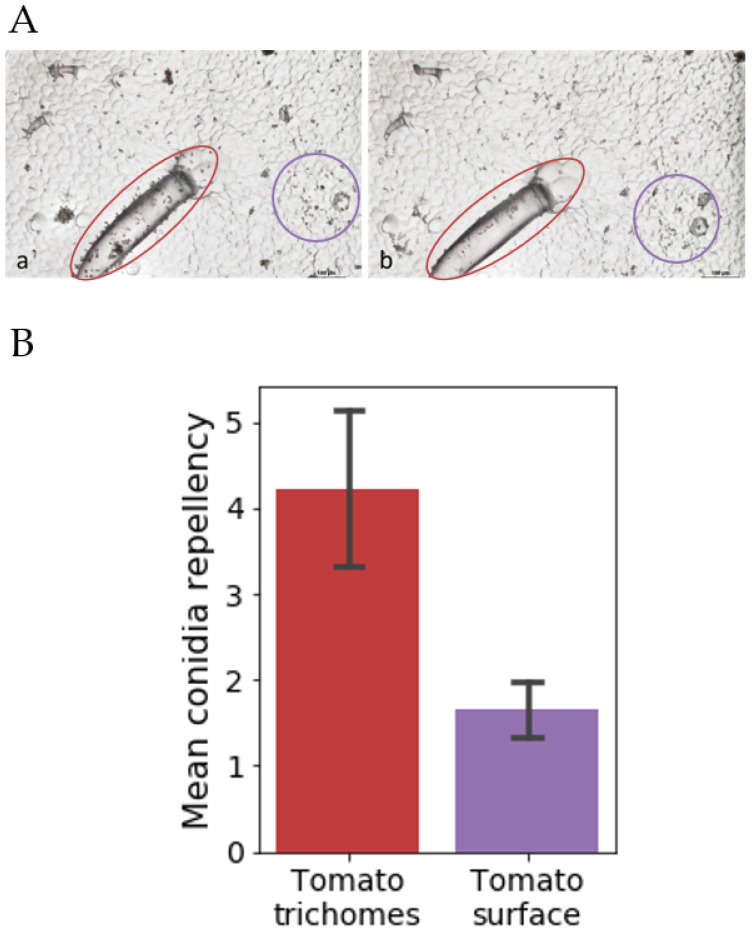
Spore repellency of tomato trichomes replica. (**A**) Microscope images of a trichome and surrounding surface of a tomato leaf replica before (**a**) and after (**b**) wash. The amount of conidia washed from the trichomes (red circles) seems larger than that washed from other parts of the surface (purple circles). (**B**) Quantification of conidia repellency from trichomes (red) and the rest of the surface (purple) replica. Conidia repellency is significantly higher in trichomes than in the rest of the replica surface.

Our microstructural replicas could not mimic the self-cleaning and superhydrophobicity of *C. esculenta* leaves. Yet, the unexpected high conidia repellency of PDMS replica of tomato leaves suggested that other microstructures might induce surface conidia repellency properties. To better understand this phenomenon, we inspected the images of the surface before and after the washing procedure and found that in hydrophobic replicas of tomato leaves, trichomes were washed more efficiently than other elements (Figure 6A). To quantify this observation, we performed a separate conidia count for trichomes and for the rest of the replica surface. This count confirmed that conidia were washed significantly more efficiently from trichomes than any other surface element in the hydrophobic tomato leaf replica (Figure 6B).

**Figure 7:**
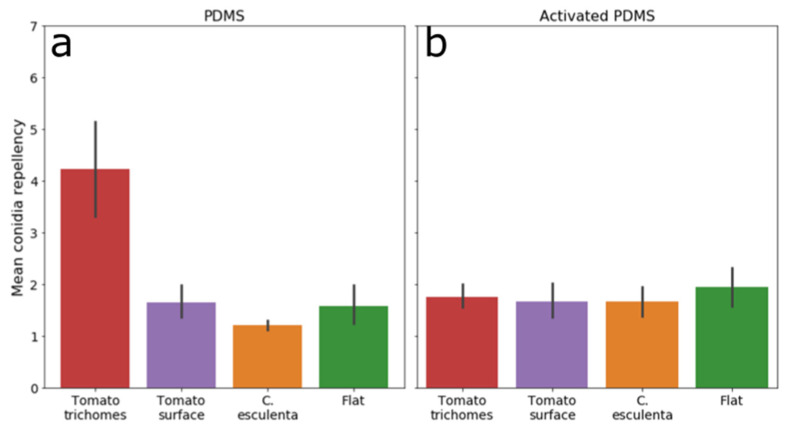
Washing efficiency of all synthetic surfaces separating trichomes from the rest of the surface. Summarization of washing efficiencies of all synthetic surfaces examined in this study. (**a**) PDMS surfaces, (**b**) Activated PDMS surfaces. Trichomes were counted separately from the rest of the tomato surface (red and purple bars, respectively). Only hydrophobic trichomes had washing efficiency significantly higher than all other surfaces.

Next, we examined the washing efficiency of PDMS replicated trichomes to the washing efficiency of all other surfaces. We counted conidia before and after wash on trichomes and the rest of the surface on activated (hydrophilic) PDMS replicas of tomato leaves and also compared the washing efficiency to that of *C. esculenta* replica and flat surfaces made from both PDMS and activated PDMS. We found that conidia attached to trichomes were washed more efficiently relatively to all other replicated surfaces examined in this study. Figure 7 demonstrates a comparison between the conidia repellency of all PDMS and activated PDMS surfaces and shows that the conidia repellency of PDMS replicated trichomes is higher than any other tested hydrophobic PDMS surface. Additionally, tomato PDMS replicas, which did not include trichomes, did not show higher conidia repellency than other PDMS surfaces, indicating that the high conidia repellency observed in PDMS tomato leaves replicas was due to the high conidia repellency of the trichomes. Among hydrophilic PDMS surfaces, no difference was observed between any of the surface structures. Thus, the trichome microstructural component demonstrated high conidia repellency abilities, but only when the surface was hydrophobic. It is important to note that *Botrytis* conidia surface is known to be hydrophobic [61], which implies different interactions between the conidia and hydrophobic or hydrophilic surfaces. These interactions can change if different fungi with different surface hydrophobicities are used. Additionally, we performed all our washes using water, and it is possible that the results will vary given a different washing liquid. Nonetheless, these results show the possibilities for a new, previously unknown microstructure that repels conidia, but only when hydrophobic. This opens up an avenue to study the mechanism involved in conidia–surface interaction, which is a complex result of both surface microstructure and hydrophobicity.

## 4. Discussion

The goal of this work was to design biomimetic, simple, and affordable surfaces with conidia repellency properties for use in agriculture, specifically post-harvest. To address this challenge, we used *B.cinerea* as a model for a pervasive pathogenic fungus and two surface models: *C. esculenta* for a leaf whose self-cleaning properties are known, and tomato—a natural host for *B. cinerea.* We tested the leaves, and their synthetic, PDMS, or activated PDMS based microstructural replications, as well as structureless PDMS surfaces for hydrophobicity and conidia washing abilities. We found that microstructure or hydrophobicity alone could not explain conidia washing ability. Additionally, we found a new microstructure in the form of tomato trichomes that could, in a hydrophobic state, be used as a conidia repellent structure.

As we tested for the proportion between conidia remaining on the surface after wash and conidia initially applied upon the surface, we were essentially looking at the adhesion process of conidia upon a surface. The common theory is that attachment of fungal conidia occurs in two distinct stages: immediate adhesion, which is characterized by relatively weak adhesive forces; and delayed adhesion, which occurs only with viable conidia and develops over time, in conditions that are suitable for germination [26]. In our study, we found no significant correlation between conidia density and surface washing efficiency, suggesting that conidia adhesion tested in this work is at the first stage, determined by physical (rather than biological) adhesion forces [62]. As such, the two surface physical properties we examined (hydrophobicity and microstructure) were expected to influence surface washing efficiency.

We used soft lithography as a replication method for biomimetics of leaf surface microstructure. Although our replication procedure replicated only the microstructure of the leaf surface, without nanostructure replication, we still found that the structure affected hydrophobicity values. Previous studies referred mostly to the nanostructure as the major component in surface hydrophobicity [63,64,65,66], but our study shows that microstructure alone also plays an important role. In this study, we found no correlation between surface hydrophobicity, as it was presented using apparent contact angle, and conidia repellency. Super hydrophobic structures are often used as biomimetic anti-biofouling and self-cleaning surfaces [23,24]. However, previous studies looking at fungal conidia adhesion to surfaces reported contradicting results of increased adhesion to hydrophobic [67,68] or hydrophilic [69] surfaces, and some have demonstrated that surface wettability did not play a major role in the adhesion of conidia [70]. Our study suggests that, in the case of *B. cinerea*, there is no correlation between surface hydrophobicity and conidia adhesion. This finding, however, may be attributed to the characteristic hydrophobicity of *B. cinerea* surface [71] and may vary when studying different fungi.

The second physical parameter we focused on was microstructure as previous studies showed that nano- or high-resolution micro-components, can successfully reduce bacterial adhesion to surfaces [27,72] and enhance self-cleaning effects [23]. In this study, the microstructural replication could not recapitulate the conidia repellency observed in the natural system. We hypothesize that this is due to the grooves of *C. esculenta* leaves replications that seemed larger than most conidia. We suspected that *B. cinerea* conidia penetrated the surface grooves and did not wash off by the water due to the hydrophobic nature of both the replication and the conidia [73]. A similar phenomenon was previously shown with bacteria [74] and with algae [28]. In future studies, other known functional structures of leaves and other organisms [48,75,76,77] can be replicated and tested for conidia repellency properties. In this study, we found one conidia repelling structure-tomato trichomes. These findings correlate with previous studies, suggesting that plant trichomes may serve not only as a chemical [78,79], but also as a structural protection mechanism [79,80,81]. This repellency was not observed in hydrophilic trichomes (trichomes on activated PDMS replications), which matches previous claims that surface structure may induce dissimilar effects on materials with different hydrophilic properties [82].

To conclude, the potential of self-cleaning structures—inspired by bio-surfaces—has been explored for decades [31,48,75,83], however there is still a lack of competitive solutions for food safety and agriculture. In addition to nano-scale structures and chemical additives, the investigation of the microstructure of natural surfaces can provide a promising option, considering a simpler manufacturing method. In the current study, the self-cleaning ability of natural leaves has been employed to understand the correlation between surfaces’ conidia washing ability, hydrophobicity, and microstructure. We found that certain microstructures might provide synthetic surfaces with conidia repellency properties under certain chemical attributes. Therefore, future studies are required to determine the optimal topographical dimensions, based on the idea that the distance between the grooves of the micro structure should not exceed the size of conidia [27]. The pathogen type considered in the study is only one example of the interaction between pathogens and surface structures. Taking into account the wide variety of shapes and properties of pathogens, other fungi may exhibit different interactions. Future studies on conidia washing driven mechanisms in the context of micro-scale surfaces may lead to more efficient self-cleaning surface microstructures.
